# Can Virus-like Particles Be Used as Synergistic Agent in Pest Management?

**DOI:** 10.3390/v14050943

**Published:** 2022-04-30

**Authors:** Caroline Deshayes, Anne-Sophie Gosselin-Grenet, Mylène Ogliastro, Bruno Lapied, Véronique Apaire-Marchais

**Affiliations:** 1 University Angers, INRAE, SIFCIR, SFR QUASAV, F-49045 Angers, France; caroline.deshayes@univ-angers.fr (C.D.); veronique.marchais@univ-angers.fr (V.A.-M.); 2 DGIMI, University of Montpellier, INRAE, F-34095 Montpellier, France; anne-sophie.gosselin-grenet@umontpellier.fr (A.-S.G.-G.); marie-helene.ogliastro@inrae.fr (M.O.)

**Keywords:** virus-like particles, synergistic agent, intracellular calcium, insecticide, pest management

## Abstract

Among novel strategies proposed in pest management, synergistic agents are used to improve insecticide efficacy through an elevation of intracellular calcium concentration that activates the calcium-dependent intracellular pathway. This leads to a changed target site conformation and to increased sensitivity to insecticides while reducing their concentrations. Because virus-like particles (VLPs) increase the intracellular calcium concentration, they can be used as a synergistic agent to synergize the effect of insecticides. VLPs are self-assembled viral protein complexes, and by contrast to entomopathogen viruses, they are devoid of genetic material, which makes them non-infectious and safer than viruses. Although VLPs are well-known to be used in human health, we propose in this study the development of a promising strategy based on the use of VLPs as synergistic agents in pest management. This will lead to increased insecticides efficacy while reducing their concentrations.

## 1. Introduction

Today, world agriculture faces a major challenge: increasing food production to feed about 9 billion people by 2050 [1]. To improve productivity, farmers still largely rely on the extensive use of chemical insecticides to control insect pests. However, the intense use of limited number of active ingredients has generated insect-selective pressure for resistance and has led to accumulation of residual effects that impact the environment and human health [2,3,4]. Regarding insecticide resistance, hundreds of insect species have developed resistance to at least one insecticide, impacting insect control [4]. Different resistance mechanisms have been described, such as insecticide target-site mutations, detoxification enzymes overproduction, penetration resistance by cuticle modification, and behavioral resistance [5,6]. The international Insecticide-Resistance Action Committee (IRAC), including stakeholders, has been developed to improve resistance awareness and Insecticide-Resistance Management (IRM) programs in crop protection [7]. To limit the side effects due to overuse of insecticides, integrated pest management (IPM) has been implemented. It is an effective and environmental approach to pest management, including the reduction of insecticides [8].

In this context, sustainable strategies have recently been developed. This includes the use of microbial pest-control agents (viruses, bacteria, and fungi) [9]; chemical mediators such as pheromones and kairomones [10]; and/or natural substances of plant, animal, or mineral origin [11]. More recently, RNA interference (RNAi)-based strategies, which do not require plant genetic modification, have been also investigated as a new method for insect pest control [12,13]. Among additional innovative strategies, nanoscale formulations of insecticides can provide controlled release of active ingredients while efficiently enhancing permeability, stability, and solubility [14,15]. However, the use of such nanopesticides in food production has raised many questions about their safety and ecotoxicological risks [16]. Consequently, the design of innovative crop-protection strategies is required for safer and more efficient treatments.

For several years, we have proposed a new patented concept based on the use of chemical synergistic agents combined with a given insecticide, which are able to optimize, in both in vitro and in vivo, the efficacy of the treatment while reducing the concentrations [17,18,19,20,21,22,23,24,25]. Compared to classical synergists (e.g., pyperonyl butoxide, S,S,S-tributyl phosphorotrithioate (DEF), diethyl maleate), which are known to inhibit detoxification enzymes involved in the hydrolysis of insecticides [26], the synergistic agents increase the sensitivity of membrane receptors and/or ion channels to the insecticide through the activation of the calcium-dependent signaling pathways following an elevation of intracellular calcium concentration. This results in the target site conformational changes involved in the increased sensitivity to insecticides [21,25]. This alternative allows to produce a stronger effect of a given insecticide used at lower concentrations than that obtained with the same molecule but used alone in a formulation at higher concentrations.

## 2. Synergistic Agents as Innovative Strategies to Improve Insecticides Efficacy

As already indicated, calcium ions play a key role on the membrane target sensitivity to insecticides. They generate versatile activation of specific calcium-dependent intracellular signaling pathways that determine a large variety of functions [27] known, for some of them, to regulate the target site conformation, which thereby modulates insecticide sensitivity [25]. Many calcium-dependent cellular and molecular factors can modulate insecticide efficacy through the activation of specific, complex signaling cascades that trigger phosphorylation/dephosphorylation process. This modulatory effect has been demonstrated in vitro with different classes of conventional insecticides, including phenylpyrazoles, neonicotinoids, pyrethroids, oxadiazines, organophosphates, and carbamates, acting on insect receptors and ion channels, such as voltage-dependent sodium channel channels (NaV), nicotinic acetylcholine receptor (nAChR), gamma-aminobutyric acid receptor (GABAR), and acetylcholinesterase (AChE) [21,24,25,28,29,30,31,32,33]. These intracellular signal transduction cascades typically amplify the calcium-dependent messages via the stimulation of effector enzymes (e.g., adenylyl cyclase, phospholipase C, guanylate cyclase), which catalyze the production or, in the case of ions, release of the second messengers (e.g., calcium ions, cAMP, cGMP, diacylglycerol, and IP3). In the last case, these second messengers are present at very low concentrations in resting insect cells and can reach relatively high concentration levels when they are stimulated. In all cases, second messengers production is controlled temporally and spatially, allowing subsequent efficient activations of kinases and/or phosphatases (e.g., protein kinase A, G, C, calcium/calmodulin-dependent protein kinase II (CaM-Kinase II), protein phosphatase (PP1/2A), calcineurin), involved in the regulation of the membrane target conformation. From these results has emerged the novel synergistic agent concept [19] based on (i) the role on intracellular calcium rise and the subsequent stimulation of calcium-dependent signaling pathways, (ii) the target site-conformational changes, and (iii) the calcium-dependent increase in target sites sensitivity to insecticides. One of the most interesting features is that the synergistic agent is a chemical or microorganism (e.g., insect viruses) that has no effect or only a limited effect itself. It indirectly optimizes the efficacy of insecticides at non-toxic concentrations through the activation of specific calcium-dependent intracellular signaling pathways that are involved in the regulation of membrane receptor and ion channel functions targeted by insecticides [19]. Among chemicals, N,N-diethyl-meta-toluamide (DEET) and insect repellent (IR3535) can be considered as synergistic agents. Previous findings performed both in vitro and in vivo have reported synergism between different synergistic agents (e.g., DEET, IR3535) and insecticides, including carbamates, oxadiazine, and neonicotinoids [18,20,21,22,23,24,28,34]. All together, these results demonstrated that chemical synergistic agents combined with a given insecticide increase (i) the sensitivity of the target site to the insecticide and (ii) the mortality rate of insects compared to that obtained with insecticides used alone.

In the same context, insect viruses can also be used as biological synergistic agents [35,36]. In this case, the insect virus is not used as a bioinsecticide (i.e., replicating within host cells) [9] but used as a synergistic agent. The interaction of the virus (e.g., baculovirus, densovirus) with the cell membrane is sufficient to produce calcium influx, associated with an elevation of the intracellular calcium concentration, which produces the activation of calcium-dependent intracellular signaling pathways [17]. Previous findings have already reported that the baculovirus *Autographa californica* *multiple nucleopolyhedrovirus* (AcMNPV) induces intracellular calcium rise through the activation of CdCl_2_-sensitive ion channels or membrane transports in the insect cell membrane. The results have clearly indicated that AcMNPV interaction with insect cell line plasma membrane increases acetylcholinesterase sensitivity for the organophosphate insecticide chlorpyrifos-ethyl through elevation of intracellular calcium concentration [36].

From our results, we then propose another alternative, which is based on the use of virus-like particles (VLPs) as a novel synergistic agent. By contrast to viruses, VLPs present some advantages since they are composed of viral structural proteins expressed in heterologous systems that assemble spontaneously but are lacking the viral genome [37,38]. VLPs have a structure similar to native virions of about 10–200 nm in diameter and can be enveloped or non-enveloped, spherical or filamentous, and composed of a single, double, or triple layers [39]. Interest in the design and production of VLPs has increased in recent years, and several applications have been developed, including vaccination, gene therapy, drug delivery, and nanotechnology.

## 3. Various Systems Used to Produce VLPs

**Figure 1:**
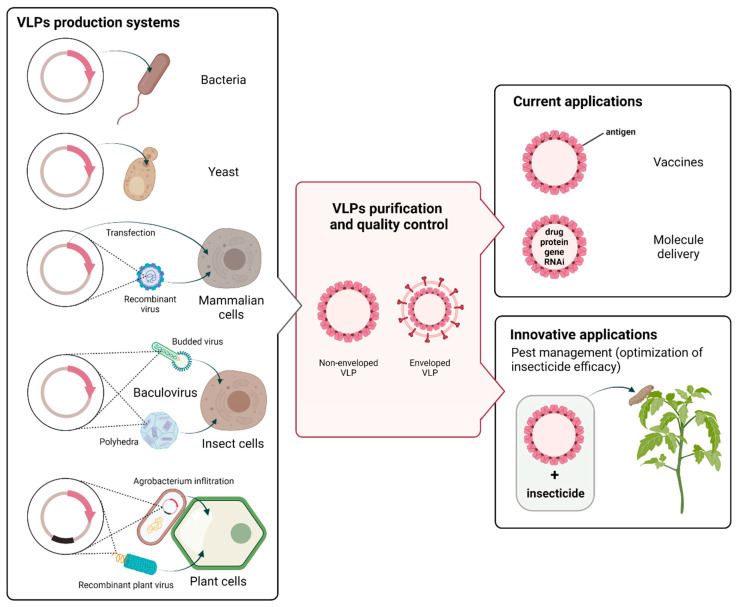
Virus-like-particles (VLPs) production systems and applications. VLPs can be produced by a variety of expression platforms, including prokaryotic and eukaryotic systems. The main eukaryotic systems include yeast, mammalian cell lines, the baculovirus/insect cell system, and plants. The production stage (left part of the figure) includes cloning of the viral structural genes of interest (in red) and introduction into the host cell by plasmid transfection or viral transduction. Depending on the method used, the transgene is integrated into the host genome or replicates in an episomal form. In plants, the rapid and transient expression of recombinant proteins is commonly based on Agrobacterium-mediated infiltration and/or plant viral vectors. After expression, the self-assembly of viral structural proteins into VLPs is highly dependent of the structure of viral proteins, the expression system, and the experimental conditions. VLPs that have similar structure as native virions can be enveloped or non-enveloped (central panel), spherical or filamentous, and composed of a single, double, or triple layer. Different downstream processing steps may be required to obtain purified VLPs without residual host contaminants. VLPs have a broad range of potential applications (right part of the figure), including vaccine production, vectors for gene therapy, and targeted drug delivery. Because VLPs are considered safer than viruses and capable of triggering the calcium concentration elevation, they could also be exploited as an alternative synergistic agent co-applied with a given insecticide for optimizing insecticide efficacy (created with Biorender.com, accessed on 3 November 2021).

VLPs can be produced in a variety of systems, including bacteria, yeast, mammalian cells, insect cells, and plants (Figure 1) [39,40,41,42,43]. VLPs can be also assembled in vitro from proteins produced in cell-free expression systems [44]. The characteristics, advantages, limitations, and yield ranges of the production systems are compared in Table 1 [41,45]. The correct folding and assembly of VLPs is a complex process, which is highly dependent on the structure of viral proteins, the expression system, and physical parameters, such as, for instance, pH, ionic strength, and temperature [39,46,47]. Three methods of cell culture are commonly used for VLPs production, including batch, fed-batch, and continuous cultivation. Technical challenges remain in the generation of VLPs in terms of yield, design, stability, and storage. Recently, chimeric VLPs have been developed. They are based on structural proteins originated from different viruses and generated through genetic fusion or chemical coupling [48,49]. Surface modifications of VLPs can be made to trigger an immune response of the host cells and/or to enhance specific recognition of VLPs by target cells [50].

### 3.1. Bacterial Cells

The expression system using *Escherichia coli* bacteria was the first established recombinant expression system. This expression system is characterized by high protein yields (Table 1), rapid cell growth, short production times, simplicity of scaling-up, and low manufacturing costs compared to eukaryotic systems. However, the inability of prokaryotic cells to undergo post-translational modifications (PTM), such as protein glycosylation and incomplete disulfide bond formation, can lead to the expression of misfolded or non-functional proteins. Bacteria can be engineered to make specific post-translational modifications, but the process then becomes expensive [52]. Furthermore, the limited solubility of recombinant viral proteins promotes the formation of inclusion bodies [51,53], and contaminant endotoxins have to be removed in the manufacturing process. Finally, protease degradation and codon bias can also contribute to lower yields. Even if *E. coli* remains the dominant bacterial strain in use, and various VLP vaccines generated using *E. coli* expression systems are under clinical trials, no VLPs are currently marketed. In addition to *E. coli,* VLPs have been successfully produced in some other bacterial species, such as *Lactobacillus casei* using a lactose-inducible promoter system for papillomavirus (HPV) L1 protein expression [77] and *Pseudomonas fluorescens* for the cowpea chlorotic mottle virus (CCMV) coat proteins expression [78]. Several chimeric VLPs vaccines have also been developed by antigen conjugation with bacteriophage Qβ RNA in *E. coli* expression platform [40].

### 3.2. Yeast Cells

This is the most popular expression system to produce VLPs is the yeast system due to its easy and high protein expression (Table 1), its ability to scale up, and the cost of production. Although yeasts provide a degree of PTM processes [41], the lack of complex PTM pathways is a major drawback of yeast expression systems. High mannose glycosylation, plasmid loss, and lower yields of protein compared to bacterial expression system remain additional issues to overcome [54]. The quality and quantity of yeast-produced VLPs are influenced by the choice of plasmid and promoter and by the ratio between different structural proteins produced. More than 30 types of VLPs have been produced in yeasts, particularly in *Saccharomyces cerevisiae,* in *Pichia pastoris,* and more recently in *Hansenula polymorpha* [41]. The yeast expression systems are generally used for generating non-enveloped VLPs. Indeed, enveloped viruses that are released through budding in mammalian cells cannot bud from yeast since the outer membrane is covered by a wall of mannoproteins and chitin [79]. However, yeast systems whose cell walls have been almost completely removed have been successfully used for HIV-1 Gag protein VLPs and Dengue virus serotype 2 VLPs [55,56]. Moreover, recent significant advances in VLPs production processes have allowed an increasing number of VLPs produced by secretion and the production of multilayered VLPs composed of more than one type of structural protein [41]. Several yeast-produced VLPs, such as papillomavirus (HPV) and hepatitis B virus (HBV) VLPs (Table 2), have already reached approval by regulatory agencies. Recently, the production of Chikungunya VLPs using the yeast *Pichia pastoris* has been reported [80].

### 3.3. Mammalian Cells

Although mammalian cells present lower yield for VLPs production compared to other systems (Table 1), they have the advantage to produce VLPs with appropriate PTMs essential for proper protein folding. Mammalian cells can be used to produce complex non-enveloped and enveloped VLPs composed of multiple structural proteins (up to five proteins) [48]. Several mammalian cell lines are suitable for VLPs production, including Chinese hamster ovary (CHO), baby hamster kidney-21 (BHK-21), human embryonic kidney 293 (HEK293), Vero cell lines, CAP-T cell line derived from human amniocytes, and East Lansing line-0 (ELL-0) [68]. The most frequently used CHO cell line, which is not derived from human, presents a lower risk of contamination by human viruses [68]. CHO cells have already been used for successful production of both dengue virus and hantavirus VLPs [92,93]. The HEK293 cell line is widely used to produce VLPs from rabies, HIV, and influenza viruses [66,67,94] (Table 2). More recently, Vero E6 cells has been used to produce stable SARS-CoV-2 VLPs as a candidate vaccine against the emerged disease COVID-19 [95].

VLPs are produced in cells that are previously either transiently or stably transfected or transduced with viral expression vectors (Figure 1). Although stable expression produces large amounts of protein, transient expression is preferred since high levels of proteins are obtained for shorter periods [66,67].

### 3.4. Baculovirus/Insect Cells

The baculovirus/insect cells system is the most commonly used expression system for large-scale production of both non-enveloped and enveloped VLPs, simple or complex, comprising up to five proteins [54]. This is a binary system consisting of a recombinant baculovirus as the vector and lepidopteran insect cells [42] (Figure 1). Baculoviruses are insect viruses not pathogenic to humans. They are easily genetically modified to express heterologous proteins in insect cells. The baculovirus AcMNPV is the most well-known virus used. Several proteins can be produced simultaneously from multiple promoters, usually under the control of the polyhedron (polh) or the p10 strong promoters [57]. In this system, VLP yields vary from 0.2 to hundreds µg/mL [48], which is similar to bacteria and yeast systems (Table 1). These high expression levels are also explained by the ability of the virus to shut off the cellular expression for the benefit of the expression of heterologous genes. The main insect cell lines used are *Sf*21 and *Sf*9 derived from *Spodoptera frugiperda* and the BTI-TN-5B1-4 cell line (High-Five™) derived from *Trichoplusia ni* [54,60]. It has been reported that High-Five™ cells have the highest heterologous protein expression yield [63,64]. Although the growth rate of insect cells is higher than mammalian cells, it is lower than yeast or bacteria. Production, therefore, requires longer times than those required for microbial systems. Culture media is more expensive compared to that used for yeast or bacteria, and production must be carried out in bioreactors. On the other hand, if the PTMs are more complex in insect cells compared to those generated in yeast and bacteria, the N-glycosylation pattern of the recombinant glycoproteins produced in this system is simpler than that of mammalian cells. Indeed, N-glycans from insect cells are not processed to terminally sialylated complex-type structures but are instead modified to a paucimannose structure [62,65]. This may result in a lower or total loss of biological function of the protein of interest, and this can be a disadvantage for some VLP applications in this production system. To solve this issue, genetic modifications of either insect cells or baculoviruses to include genes encoding N-glycosylation functions have been considered [42,58,59]. One of the main drawbacks of the baculovirus/insect cells system remains the contamination with the baculovirus particles that are also produced at the same time as VLPs [61]. To limit expensive purification steps, non-replicative baculoviruses have been developed to minimize the contamination [96]. Alternative insect cell line that avoids the use of baculovirus, such as Drosophila Schneider line 2 (S2 cells), can also be used to produce VLPs [60,97].

### 3.5. Plant Cells

Plant expression systems for VLPs production allow the production of properly folded complex proteins and represent a cost-effective eukaryotic system [60] that is easy to scale-up and free of mammalian pathogens. Advances in plant biotechnology have made possible the use of transgenic plants as alternatives to cell culture systems [73]. VLPs can be produced in a variety of plant species, including *Solanum tuberosum* (potato), *Lycopersicon esculentum* (tomato), *Glycine max* (soybean), *Lupinus luteus* L. (Lupin callus), *Arabidopsis thaliana,* and *Nicotiana benthamiana* or *Nicotiana tabacum* L. (tobacco) [70]. Plants can transiently or stably express viral proteins in the nucleus or in the chloroplast. PTMs can be performed on the viral proteins expressed from the nucleus, while chloroplast transformation enables high levels of transgene expression (up to 80% of total soluble proteins) [74] but lacks PTMs [60]. Stable expression is time-consuming and can lead to low expression yields [72]. On the other hand, transient expression obtained through either Agrobacterium infiltration or plant viral vectors is easy, quick, and highly productive [71] (Figure 1). Transient transgenic plants also have the advantage of not being classified as genetically modified organisms (GMO).

Advantages provided by transgenic plants are the low cost of production, estimated to be 10 to 50 times lower than products derived from *E. coli* and 140 times lower than production using baculovirus-based insect cells [69,76], and the simple scaling-up that requires few materials except for cultivation surface. Since the plants are edible, they could also serve as delivery mode for oral vaccination, thereby reducing the purification costs. Plants also present advantages in terms of storage for recombinant proteins that are protected within plant tissue. The main limitations are the long timeline for transgenic plants establishment, the low expression levels, and antigen degradation during in vivo delivery [48]. Two replicon systems in particular have been recently developed to induce strong expression of VLPs in plants: the deconstructed viral vectors composed of tobacco mosaic virus RNA replicon system (MagnICON) and the Geminiviral BeYDV DNA replicon system [98,99]. These replication systems have led to an increase of more than 80-fold in the accumulation of Norwalk virus (NV) capsid protein VLPs in transgenic tobacco and tomato [100]. Finally, both enveloped and non-enveloped as well as native and chimeric plant-derived VLPs have been produced [75]. Chimeric VLPs are composed of plant viral vectors, such as tobacco mosaic virus (TMV), cucumber mosaic virus (CMV), alfalfa mosaic virus (A1MV), cowpea mosaic virus (CPMV), papaya mosaic virus (PapMV), and the potato X virus (PVX) that carry recombinant proteins (viral or not). This strategy has enabled the production of over 100 experimental plant virus-based vaccines against a wide range of diseases in both humans and animals [101,102,103].

## 4. Current Applications of VLPs

Up to date, VLPs are currently used for vaccination and are in development as delivery systems for antigens, genes, nucleic acids, or drugs [37,82,104].

### 4.1. VLP-Based Vaccines

New generations of vaccines improve safety by using viral proteins, VLPs, or nucleic acids. Having no genomic material, VLPs have an advantage compared to classical vaccines. They can present viral epitopes via repetitive and highly organized structures, such as those expressed in natural infectious virions, without any risk of infection. VLPs interact with various components of the immune system to produce strong immune responses [37]. In addition, VLPs can have adjuvant properties [37]. There are different approaches to develop VLP-based vaccines: VLP vaccines that mimic the natural virus and chimeric VLPs (i.e., encapsulating the antigen or displaying the antigen on the exterior) [37].

Several VLP-based vaccines are already available with good results, and many others are in clinical trials or research stages [45,81]. VLPs-based vaccines that have been marketed for use in humans include recombinant vaccines for hepatitis B virus, human papillomavirus, and hepatitis E virus and in the veterinary field against porcine circovirus type 2 (Table 2). Several VLPs are under clinical trial as potential vaccines for influenza, Epstein Barr virus, and malaria and against emerging viral infections such as Ebola, avian flu, MERS, and SARS-CoV [105]. VLPs provide a promising approach for developing safe and effective flavivirus vaccines against Zika, Dengue, West Nile, and Japanese encephalitis viruses [106].

Specific vaccine antigens were generated by different expression systems to induce protective immune responses (Table 2). For example, vaccines against hepatitis B virus are produced in a yeast system to express stable HBsAg VLPs (i.e., Engenerix^®^) or in mammalian cells (CHO) to express three HBV antigens (S, pre-S1, and pre-S2), leading to higher immunogenicity (Sci-B-Vac^®^). The hepatitis E VLP-based vaccine, Hecolin^®^, only licensed in China, is produced using *E. coli* expression system, and another vaccine expressed in insect cells is under clinical trial (Table 2) [81]. Concerning the malaria vaccine Mosquirix^®^, enhanced efficacy was obtained with a yeast-produced direct fusion between HBsAg and CSP antigen. In this system, HBsAg is only used as a carrier matrix for the malaria antigen and does not induce antibodies against HBsAg [107].The prophylactic vaccination against HPV is based on VLPs produced by introducing the L1 gene encoding a capsid protein into eukaryotic cells (insects or yeasts). Licensed human and veterinary vaccines are based on artificial VLPs derived from human or animal viruses. However, plant virus-derived platforms can be used for the creation of novel vaccines [108]. HBV- and norovirus-derived VLPs are the most studied VLPs, produced in plant-based systems by three different species of transgenic plants: *S. tuberosum* [109], *L. esculentum* [70], and *N. benthamiana* [70,75,110]. Many plant virus-derived nanoparticles have also been tested as antigen carriers for different human or animal vaccines against influenza virus, hepatitis C virus, Japanese encephalitis virus, canine parvovirus, and classical swine fever virus [108]. Plant VLP vaccines are produced in the different expression systems already described, such as *E. coli,* and cloned in commercial plasmid, yeast, insect cells, mammalian cells, or the most commonly used plants *N. tabacum, N. benthamania,* and *A. thaliana* [43,45,108]. For influenza viruses, plant-derived VLPs are an alternative to the currently available manufacturing platforms for seasonal vaccines, and a seasonal recombinant quadrivalent VLP vaccine is currently in clinical trial phase [111] (Table 2).

### 4.2. Molecule Delivery

For a few decades, nanoparticle (NP)-based delivery agents, such as liposomes, polymers, dendrimers, magnetic nanoparticles, and protein-based NPs, have been used as carriers for drug delivery. These systems comprise the administration and controlled-release delivery of pharmaceutical compounds to a specific area in tissues, improving efficacy and safety [112]. Emerging research focuses on the development of protein-based NPs derived from viral capsids (VLPs) as targeted therapeutic delivery agents [39,113,114] (Table 2). Indeed, VLPs can pack and deliver therapeutic cargo such as chemotherapeutic drugs, nucleic acids, proteins, and peptides. Different strategies have been developed to carry the cargo either inside or outside the VLPs: (i) viral capsids disassembly by altering pH and buffer conditions and reassembly to encapsulate the desired cargo using buffer exchange methods, (ii) infusion of cargo due to changes in pH and salt concentrations, (iii) genetic engineering techniques utilizing genetically conjugated scaffolding proteins to encapsulate drugs, and (iv) bioconjugation using exterior surface-exposed residues [37].

Compared to classical NPs, VLPs present several advantages, including specific cell targeting. VLPs show a natural tropism to certain tissue, and a more specific targeting function can be obtained through attaching receptor-recognizing domains to the drug carriers [115] (Table 2). For example, the tumor-targeting peptide (RGD) inserted through genetic modification into the major immunodominant loop region of HBc (hepatitis B core protein) VLPs enhanced tumor-homing in mice model [116]. Furthermore, cell targeting has been achieved through functionalization of the exterior of the bacteriophage MS2 capsids with the cell penetrating HIV-1 Tat peptide [117]. In addition to specific targeting, VLPs permit efficient host cell penetration, biocompatibility, and degradability [81]. VLPs, like original viruses, have the ability to escape the endosome before lysosomal degradation occurs [115]. Indeed, after interaction with viral receptors, most viruses utilize the endocytosis pathways (clathrin-mediated endocytosis, caveolae-mediated endocytosis, and macropinocytosis) to enter cells efficiently [118]. Once endocytosed, viruses can escape from late endosomes or lysosomes and release their contents to cytoplasm by different pathways, such as membrane pore formation, membrane fusion, membrane penetration, and membrane disruption. VLPs can be engineered to display cell-penetrating peptides to aid in escaping the endosome, enhancing the delivery of functional therapeutic cargo [114]. For example, MS2 VLPs have been modified with a histidine-rich fusogenic peptide (H5WYG) to promote endosomal escape of internalized VLPs. This drug delivery system is under research for delivery of doxorubicin, cisplatin, 5-fluorouracil, and siRNA for hepatocellular carcinoma treatment [91] (Table 2).

## 5. Future Direction in Pest Management: VLPs as Synergistic Agent of Insecticides

**Figure 2:**
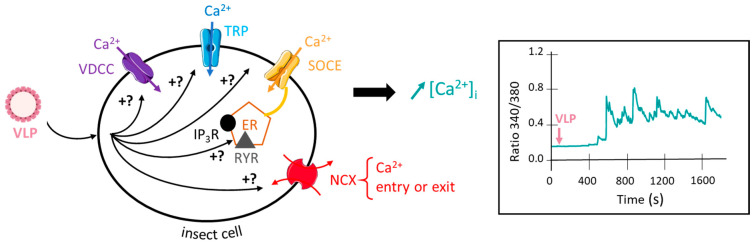
VLPs induce a multicomponent intracellular calcium rise. The scheme summarizes the hypothetic mechanisms by which VLPs increase intracellular calcium concentration. Inset: Representative multicomponent effect of VLPs on intracellular calcium concentration in Fura-2 loaded isolated insect neuron cell body using the calcium imaging ratiometric method (C. Deshayes, unpublished data). VDCC, voltage-dependent calcium channel; TRP, transient receptor potential channel; SOCE, store-operated calcium entry; NCX, sodium–calcium exchanger; RYR, ryanodine receptor; IP3R, inositol triphosphate receptor; ER, endoplasmic reticulum.

Up to date, VLPs are not used in the context of pest management. However, VLPs have recently received our attention because (i) they have been used worldwide in human health for over 30 years for HBV vaccination with a safety and efficacy profile more than satisfactory, taking into account the available safety data [119,120,121], and (ii) they can produce multiphasic elevation of intracellular calcium concentration in insect cells, as shown in the graph obtained by calcium imaging ratiometric method (data from our laboratory, *inset* Figure 2). Like classical synergistic agents [19], VLPs induce multiphasic components of the calcium elevation, suggesting the involvement of multiple molecular cellular and intracellular events (Figure 2). This includes plasma membrane calcium channel and/or receptors (e.g., voltage-dependent calcium channels, transient receptor potential (TRP), store-operated calcium entry (SOCE), and intracellular receptors (ryanodine receptor (RYR), inositol 1,4,5-trisphosphate-receptor (IP3R)) that participate in calcium rise through calcium-induced calcium release (CICR) or calcium release-activated channels (CRAC) mechanisms [122]. As stated above, the increase in intracellular calcium concentration is the essential prerequisite for producing an optimization of insecticide efficacy while reducing the concentrations used. Preliminary toxicological in vivo studies performed on the aphid *Acyrthosiphon pisum* revealed that the mortality rate (using an artificial diet bioassay and measured at 48 h) produced by the insecticide indoxacarb is increased by about 25% when co-applied with VLPs (5 × 10^9^ particles/µL) (C. Deshayes, unpublished data). VLPs are considered safer than viruses due to the absence of the virus genome and capability of triggering calcium concentration elevation. For these reasons, they could be proposed as alternative synergistic agents co-applied with a given insecticide for optimizing insecticide efficacy through the elevation of the intracellular calcium concentration and be exploited in pest management. Nevertheless, ecotoxicological and environmental stability studies should be required before further applications.

## Figures and Tables

**Table 1:** Characteristics, advantages, and disadvantages of the different VPLs platforms (modified with permission from Ref. [45], Copyright 2017 Elsevier B.V. license number 5296930551852).

Production Platforms	Advantages	Disadvantages	Yield Range	Type of VLP Produced	References
*E. coli*	Ease of expression Fast growth rate High-level expression Simple process scale-up Low production cost	No PTMs Limited protein solubility Misfolded proteins Endotoxin contamination Production of simple VLPs	0.75 to 700 μg/mL	Non-enveloped	[45,51,52,53]
Yeast	Capacity of simple PTMs Ease of expression High-level expression Ability to scale-up Low production cost	Limited PTMs Risk of incorrect folding and assembly Production of simple VLPs (cell wall)	0.75 to 700 μg/mL	Non-enveloped; Enveloped (if using yeast spheroplasts); Unique and multiple structural proteins	[41,45,54,55,56]
Insect cells	Capacity of most eukaryotic-type PTMs Cell culture in suspension, without CO_2_ Free of mammalian pathogens Production of complex VLPs	Simpler N-glycosylation compared to mammalian cells Low yield High production cost Difficult to scale-up Baculovirus contamination	0.2 and 18 μg/mL	Non-enveloped; Enveloped; Unique and multiple structural proteins	[42,45,48,54,57,58,59,60,61,62,63,64,65]
Mammalian cells	Complex PTMs Assembly and folding Possible cell culture in suspension Production of complex VLPs	Low cell growth rate Long expression time Low yield High production cost Difficult to scale-up Risk of contamination by mammalian pathogens	0.018 and 10 μg/mL	Non-enveloped; Enveloped; Unique and multiple structural proteins	[45,48,66,67,68]
Plants	Complex PTMs (nucleus) Ease of expression High expression levels of up to 80% total soluble protein Ability to scale-up Low production cost VLPs storage (protected in plants) Potential oral immunization by simply ingesting VLPs in edible plant parts	No PTMs (chloroplasts) Time-consuming production of stable transgenic plants Low-level expression Low VLP assembly and stability Production of simple VLPs Technical issues (transgenic plants)	4 to 2380 pg/mg of leaf	Non-enveloped; Unique and multiple structural proteins	[45,60,69,70,71,72,73,74,75,76]

**Table 2:** Non-exhaustive applications of VLP in vaccines or molecule delivery.

	Pathology	VLPs Composition	VLP Type	Expression System	Status	References
VACCINES	Hepatitis B virus (HBV) infection	HBsAg	NE	Yeast (*S. cerevisiae*)	Licensed (Engenerix-B^®^ and Recombivax HB^®^)	[45,81,82]
		S, pre-S1, and pre-S2		Mammalian cells (CHO)	Licensed (Sci-B-Vac^®^)	
	Human papillomavirus (HPV) infection	HPV 6/11/16/18 L1	NE	Yeast (*S. cerevisiae*)	Licensed (Gardasil^®^)	[45,69,81,82]
		HPV 6/11/16/18/31/33/45 /52/58 L1		Yeast (*S. cerevisiae*)	Licensed (Gardasil 9^®^)	
		HPV 16/18 L1		Baculovirus/Insect cells (High-Five™)	Licensed (Cervarix^®^)	
	Hepatitis E virus (HEV) infection	p239	NE	Bacteria (*E. coli*)	Licensed (China) (Hecolin^®^)	[81,82,83]
		peptide		Baculovirus/Insect cells (*Sf*9)	Clinical trial phase	
	Malaria	CSP into the HBsAg	NE	Yeast (*S. cerevisiae*)	Licensed (Mosquirix^®^)	[45,81]
	Human immunodeficiency virus (HIV) infection	p17 and p24	E	Yeast (*S. cerevisiae*)	Clinical trial phase	[45,84]
		Gag or Env		Mammalian cells (HEK293)		
		Gag or Env		Baculovirus/Insect cells (High-Five™)		
	Human parvovirus B19 infection	VP1 and VP2	NE	Baculovirus/Insect cells (*Sf*9)	Clinical trial phase	[45,82]
	Influenzavirus A infection	HA quadrivalent	E	Baculovirus/Insect cell (*Sf*9)	Licensed (Supemtek^®^)	[43,45,69,85]
				Plant (*Nicotiana benthamania*)	Clinical trial phase	
	SARS-CoV infection	SP, EP, MP	NE	Baculovirus/Insect cells (*Sf*9)	Clinical trial phase	[37,85,86]
				Plant (*Nicotiana benthamania*)	Clinical trial phase	
	Porcine circovirus type 2 infection	ORF2	NE	Baculovirus/Insect cells (*Sf*9)	Licensed (Circumvent^®^)	[69,87]
MOLECULE DELIVERY	Cancers	Bleomycin cross-linked at the surface of Dd-Ad3 VLPs	NE	Baculovirus/Insect cells (High-Five™)	In vitro research	[88]
	Hepatocellular carcinoma (HCC)	Cap structure analog or Doxorubicin cross-linked at the surface of Dd-Ad3 VLPs	NE	Baculovirus/Insect cells (High-Five™)	Preclinical research	[89]
	Systemic lupus erythematosus	miRNA-146a packaged into conjugated MS2 bacteriophage capsid coated with HIV Tat47-57 peptide	NE	Bacteria (*E. coli*)	Preclinical research	[90]
	Hepatocellular carcinoma (HCC)	Doxorubicin, cisplatin, 5-fluorouracil, or SiRNA packaged into MS2 bacteriophage capsid coated with SP94 targeting or histidine-rich fusogenic peptides	NE	Bacteria (*E. coli*)	In vitro research	[91]

CHO, Chinese hamster ovary; CSP, circumsporozoite protein; Dd-Ad3, Dodecadron derived from Adenovirus serotype 3; E, enveloped; EP, envelope; HA, hemagglutinin; HBsAg, hepatitis B surface antigen; HEK293, human embryonic kidney 293; MP, membrane; NE, non-enveloped; p, protein; *Sf*, *Spodoptera frugiperda*; SP, spike.
